# Physical fitness in community‐dwelling older adults is linked to dietary intake, gut microbiota, and metabolomic signatures

**DOI:** 10.1111/acel.13105

**Published:** 2020-01-22

**Authors:** Josué L. Castro‐Mejía, Bekzod Khakimov, Łukasz Krych, Jacob Bülow, Rasmus L. Bechshøft, Grith Højfeldt, Kenneth H. Mertz, Eva Stahl Garne, Simon R. Schacht, Hajar F. Ahmad, Witold Kot, Lars H. Hansen, Federico J. A. Perez‐Cueto, Mads V. Lind, Aske J. Lassen, Inge Tetens, Tenna Jensen, Søren Reitelseder, Astrid P. Jespersen, Lars Holm, Søren B. Engelsen, Dennis S. Nielsen

**Affiliations:** ^1^ Department of Food Science University of Copenhagen Frederiksberg C Denmark; ^2^ Department of Orthopedic Surgery M Bispebjerg Hospital Copenhagen NV Denmark; ^3^ Department of Biomedical Sciences University of Copenhagen Copenhagen N Denmark; ^4^ Department of Nutrition, Exercise and Sports University of Copenhagen Frederiksberg C Denmark; ^5^ Faculty of Industrial Science and Technology Industrial Biotechnology Program Universiti Malaysia Pahang Pahang Malaysia; ^6^ Department of Plant and Environmental Sciences University of Copenhagen Frederiksberg C Denmark; ^7^ Copenhagen Center for Health Research in the Humanities The SAXO Institute University of Copenhagen Copenhagen SV Denmark; ^8^ School of Sport, Exercise and Rehabilitation Sciences University of Birmingham Birmingham UK

**Keywords:** aging, energy and dietary fiber intake, gut microbiota, host metabolome, physical fitness, proinsulin‐C‐peptide

## Abstract

When humans age, changes in body composition arise along with lifestyle‐associated disorders influencing fitness and physical decline. Here we provide a comprehensive view of dietary intake, physical activity, gut microbiota (GM), and host metabolome in relation to physical fitness of 207 community‐dwelling subjects aged +65 years. Stratification on anthropometric/body composition/physical performance measurements (ABPm) variables identified two phenotypes (high/low‐fitness) clearly linked to dietary intake, physical activity, GM, and host metabolome patterns. Strikingly, despite a higher energy intake high‐fitness subjects were characterized by leaner bodies and lower fasting proinsulin‐C‐peptide/blood glucose levels in a mechanism likely driven by higher dietary fiber intake, physical activity and increased abundance of Bifidobacteriales and Clostridiales species in GM and associated metabolites (i.e., enterolactone). These factors explained 50.1% of the individual variation in physical fitness. We propose that targeting dietary strategies for modulation of GM and host metabolome interactions may allow establishing therapeutic approaches to delay and possibly revert comorbidities of aging.

## Graphical abstract

Physical fitness among older subjects is characterized by variations in glycemic regulation in a mechanism likely driven by energy and dietary fiber intake, differential abundance of Bifidobacteriales and Clostridiales species in GM and their associated metabolites (i.e., enterolactone). Several signatures seen in frail older adults are already evident in nonfrail, pointing at the importance of early intervention strategies also in this age group.

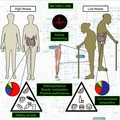

## 1 INTRODUCTION

Throughout the course of aging, physical impairment and changes in body composition may arise along with a number of lifestyle‐associated disorders influencing physical decline and ultimately frailty (Holm et al., 2014; Xue, 2011). Aging inevitably occurs in all organisms with genetics, epigenetics, and environmental exposures (e.g., diet, physical activity) being modulators of the bodily deterioration caused by biological age (Khan, Singer, & Vaughan, 2017). A number of guidelines toward dietary and daily physical activity recommendations are currently available; however, adherence remains a significant challenge (Gopinath, Russell, Kifley, Flood, & Mitchell, 2016). Further, food perception and dietary habits can be strongly altered during the course of life, particularly those traits associated with the loss of appetite (declined senses of smell and taste), occurrence of immune‐senescence and deterioration of the gastrointestinal system (Giezenaar et al., 2016).

During the last decade, the gut microbiota (GM) has been recognized as a signaling hub that integrates dietary habits with genetic and immune signals throughout life (Thaiss, Zmora, Levy, & Elinav, 2016). Many inflammatory and metabolic disorders, such as obesity, diabetes, and inflammatory reactions, are linked with GM dysbiosis (Boulangé, Neves, Chilloux, Nicholson, & Dumas, 2016). Among Irish older subjects, frailty has been linked with changing GM signatures (Claesson et al., 2012) and age‐related insulin resistance has been found to be regulated by the metabolic activity (e.g., production of short‐chain fatty acids—SCFA) of a number of Clostridiales species (e.g., *Clostridium IV*, *Ruminococcus, Saccharofermentans*) and *Akkermansia muciniphila* (Biagi et al., 2010; Bodogai et al., 2018; Kong et al., 2016). Further, low abundance of these bacteria leads to increased leakage of pro‐inflammatory epitopes from the gut to the bloodstream (due to leaky gut syndrome) activating monocytes inflammation and subsequently impair insulin signaling in rodents (Bodogai et al., 2018).

It is well‐established, that frail older adults are characterized by changed dietary habits and altered GM and metabolic signatures relative to nonfrail peers (Claesson et al., 2012; Lustgarten, Price, Chalé, & Fielding, 2014), but whether similar signatures can be identified among nonfrail older adults of different physical capacity has, to the best of our knowledge, not been investigated previously. A few studies have focused on frail individuals showing that a reduced consumption of dietary fiber compromises the GM associated production of SCFA required for maintenance of colonic epithelial cells and regulation of immune and inflammatory responses (Biagi et al., 2010; Claesson et al., 2012; Kong et al., 2016). Likewise, GM signatures were found to correspond with frailty indexes in a large cohort of older adults, whose GM composition were inherently driven by dietary patterns (Claesson et al., 2012). Moreover, metabolites related to GM metabolism (e.g., p‐cresol sulfate, indoxyl sulfate), peroxisome proliferator‐activated receptors‐alpha activation, and insulin resistance likely influence physical function in physically impaired older adults (Lustgarten et al., 2014).

Understanding how dietary intake and physical activity in nonfrail older adults alter the GM–metabolome axis, and ultimately the physical fitness and the risk of functional decline, is of great clinical interest for the affected subjects as well as for the society. Furthermore, identifying key components of such multifactorial processes may open opportunities to therapeutically address and possibly treat and prevent the comorbidities of aging (Khan et al., 2017). Based on this framework, we characterized dietary intake, daily physical activity, GM, and host metabolome in order to be able to explain physical fitness of nonfrail older subjects. To this end, we included 207 individuals (65+ years old, self‐supportive and apparently healthy) recruited through the Counteracting Age‐related Loss of skeletal Muscle mass (CALM) study (http://calm.ku.dk) (Bechshøft et al., 2016). Our findings demonstrate that physical fitness and function corresponded to signatures of fasting proinsulin and average blood glucose, and characterized by clear differences in energy and dietary fiber intake, daily physical activity as well as differential abundance of GM members and a number of fecal and plasma metabolites.

## 2 RESULTS

### 2.1 Participants inclusion

Two hundred seven individuals with body mass index (BMI) ranging between 18.5 and 37.3 kg/m^2^ (Table 1) were included in this cross‐sectional study (Bechshøft et al., 2016). Subjects are representatives of community‐dwelling, self‐supportive and apparently healthy older adults living in the Danish Capital Region. Detailed inclusion criteria have been described previously (Bechshøft et al., 2016). From each individual, anthropometric, body‐composition and physical performance measurements (ABPm), average daily physical activity, dietary intake and preferences, GM composition, clinical biomarkers, as well as fecal and plasma metabolome data were obtained adding up to 1,232 analyzed features per subject (Figure S1a).

**Table 1:** Description of the study participants

Number of Participants (*n*)	207
Sex	
Men: Women	109:98
Age (y) Mean ± *SD*	70.2 ± 3.9
BMI (kg m^2^) Mean ± *SD*	25.7 ± 3.8
BMI < 25	105
BMI ≥ 25 < 30	75
BMI ≥ 30	27
HbA1c (mmol/mol)	
<39 mmol mol^−1^ (<5.7 ABG – mmol/L)^a^	167
39–46 mmol mol^−1^ (5.7–6.4 ABG – mmol/L)	40

a HbA_1c_ values above 47 mmol/mol (6.5 mmol/L average blood glucose—ABG) is a criterion for diagnosis of T2D (Gardner & Shoback, 2011).

### 2.2 Stratification of subjects according to physical fitness and activity monitoring

Participants were stratified based on noncollinear ABPm variables (Table S1; Variance Inflation Factor, VIF < 2, *r‐*coefficient < .5) into high‐ and low‐physical fitness phenotypes (level of physical capacity). These included chair‐rise test [30 s‐test]), BMI, and Dual‐energy X‐ray Absorptiometry (DXA) scans for body composition (given by leg‐soft‐tissue fat% (LST%)), determined as described previously in Bechshøft et al. (2016).

**Figure 1:**
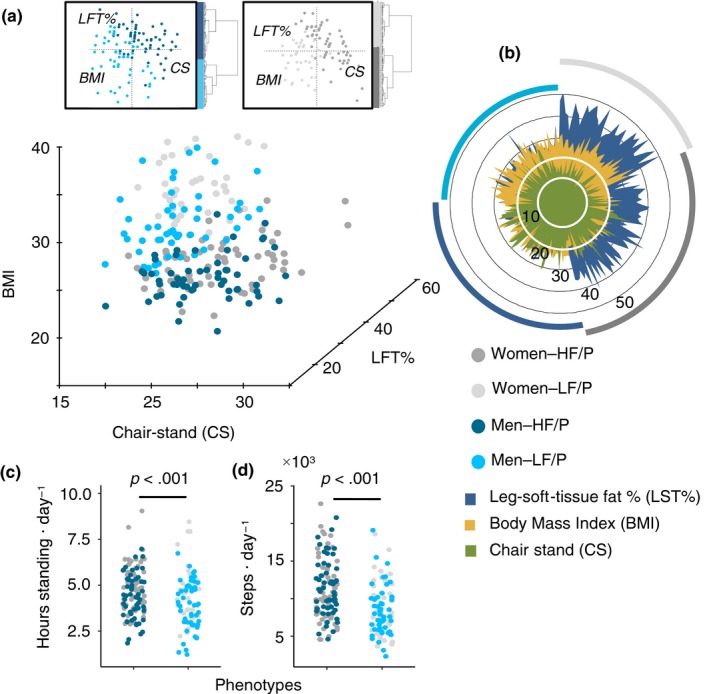
Stratification of fitness phenotypes. (a) Stratification of subjects (*n* = 207) by hierarchical clustering analysis of principal components analysis (HCA‐PCA). Stratification data matrix: [obj × vars] = [207 × 3]. HCA‐PCA was performed within sexes and based on ABP measurements. HF/P: high‐fitness (*n* = 116) and LF/P: low‐fitness phenotypes (*n* = 91). (b) ABP measurements distribution among phenotypes and sexes. (c) 4‐day activity monitoring displaying hours standing and steps on daily basis for both phenotypes. 4‐day activity data matrix: [obj × vars] = [196 × 2]

For stratification, hierarchical clustering analysis of principal component analysis (HCP‐PCA) within sexes was used to determine two fitness phenotypes [high (HF) (*n* = 116) and low (LF) (*n* = 91) (Figure 1a,b, Table 2)]. To this end, physical fitness was not defined as an outcome, but instead used as a reference to generalize physical performance within the study participants. All participants outperformed the suggested ranges for frailty according to the chair‐rise test (Guralnik et al., 1994), while LF phenotypes on average had BMI ranges categorized as overweight (WHO, 2000), as well as a greater deposition of fat mass in their legs (Figure 1b, Table 2). No significant differences (chi‐squared *p* > .08) in type of medication (e.g., blood pressure lowering and statins, see methods) or dietary supplements were determined between the two fitness phenotypes.

**Table 2:** Within sex summary of ABP measurements used for stratification of phenotypes

Functional Parameter	HF/P	LF/P	*p‐*value^a^	Refer. range	Ref. age
Women					
30 s Chair‐stand test	20.6 ± 5.0	15.7 ± 3.1	<.001	10–16^b^	65−74 years^b^
BMI	22.4 ± 2.1	28.9 ± 3.3	<.001		
LST%	35.2 ± 4.0	42.7 ± 4.6	<.001		
Men					
Chair‐rise test	22.9 ± 4.4	18.3 ± 3.9	<.001	12–18^b^	65−74 years^b^
BMI	24.0 ± 2.2	28.3 ± 3.1	<.001		
LST%	20.3 ± 3.4	27.0 ± 3.5	<.001		

Abbreviations: HF/P, high‐fitness phenotypes; LF, low‐fitness phenotypes.

a Comparison between phenotypes was performed by two‐tailed Student's *t* test.

b Reference (Guralnik et al., 1994).

In relation to daily physical activity, 4‐day activity monitoring (Dowd, Harrington, & Donnelly, 2012) showed significant differences (*p* < .001) between the two phenotypes. Longer standing periods (Figure 1c; HF mean: 4.6 ± 1.3, LF mean: 4.2 ± 1.5) and a greater number of steps per day (Figure 1d; HF mean: 11,129 ± 3,861, LF mean: 8,814 ± 3,595) were recorded among HF phenotypes. The habitual daily activity for LF phenotypes was found to be within recommended ranges (taking approximately 7,000–10,000 steps/day (Tudor‐Locke et al., 2011)), resembling the average of the adult Danish population (8,311 ± 3,125 steps/day, age of 18–75 years) (Matthiessen, Andersen, Raustorp, Knudsen, & Sørensen, 2015), and markedly outperformed by HF subjects (Figure 1d).

### 2.3 Dietary food intake in relation to fitness‐state

Using 3‐day weighted food records (3d‐WFR) (Schacht et al., 2019), the daily average energy and macronutrients intake were quantified. On average, the energy intake per person was 24.5 ± 7.4 (range of 11.5–55.2) Cal kg body weight^−1^ day^−1^. Protein contributed less of the energy intake (18.9% ± 4.1, range 9%–36%) compared to the average energy intake of fat (36.7% ± 7.3, 22%–64%) and carbohydrates (44.4% ± 7.7, 17%–66%) expressed as percentage of total energy intake.

**Figure 2:**
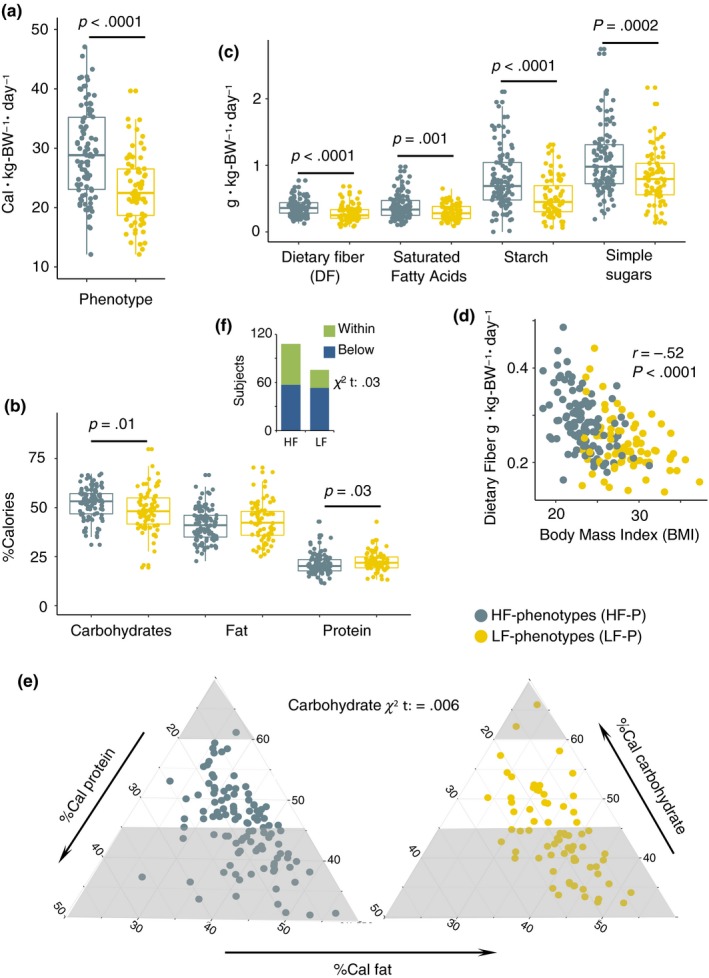
Dietary intake and distribution. (a) Total energy consumption per kg‐body‐weight per day (Cal kg body weight^−1^ day^−1^). (b) Distribution of Calories proportionally obtained from macronutrients intake in HF and LF phenotypes. (c) Intake of carbohydrates by quality and saturated free fatty acids (g kg body weight^−1^ day^−1^). (d) Pearson correlation between dietary fiber (g kg body weight^−1^ day^‐1^) and BMI depicted according to phenotypes category. (e) Proportion of subjects complying with recommended carbohydrates distribution ranges. The gray areas correspond to nonrecommended ranges as suggested by the Nordic Nutrition Recommendations. (f) Proportion of subjects complying with recommended distribution ranges of dietary fiber according to the Nordic Nutrition Recommendations. Dietary data matrix: [obj × vars] = [181 × 11]

Total energy consumption per kg body weight (Figure 2a) differed significantly (*p* < .001) between phenotypes, with an average daily intake of 29.3 Cal kg body weight^−1^ day^−1^ in HF phenotypes versus. 23.1 Cal kg body weight^−1^ day^−1^ in LF phenotypes. The higher energy intake among HF subjects was reflected in a larger fraction of energy (expressed as % energy) from carbohydrates (*p* = .01) as compared to that of dietary protein (Figure 2b and Figure S1b). The same pattern was also observed across daily average intake (g kg body weight^−1^ day^−1^) of dietary fiber (*p* < .0001), starch (*p* < .0001), simple sugars (*p* = .0002), and saturated fatty acids (*p* = .0001) (Figure 2c). Moreover, significant (*p* < .0001) negative correlations between BMI with dietary fiber consumption (*r* = −.52) (Figure 2d) energy intake (*r* = −.52), starch (*r* = −.35) and simple sugars (*r* = −.35), as well as positive associations between chair‐stand test and energy intake (*r* = .25) were found (Figure S1c–f). Questionnaires on food choices showed that HF subjects to a higher degree than LF subjects consider healthy food as an important element of their daily life (Figure S1g).

A considerable proportion of subjects from both phenotypes did not comply with the recommended minimum proportion of energy obtained from carbohydrates (Figure 2e) and dietary fiber intake (Figure 2f) as established by the Nordic Nutrition Recommendations (Nordic Council of Ministers, 2012). Yet, the frequency of compliers to noncompliers was significantly higher (carbohydrates: *p* = .006, dietary fiber: *p* = .03) in HF individuals. Furthermore, using the Goldberg cutoff (Black, 2000), 46 under‐reporters (UR) and two over‐reporters (OR) of energy intake were identified. Nonetheless, if excluded, individuals with higher physical capability (HF phenotype) still had a higher energy (*p* < .001) and energy from carbohydrates (*p* < .06) intake as compared to LF subjects (Table S2). Since UR and OR subjects did not change the overall findings, they were not excluded in downstream analyses.

### 2.4 Characterization of GM and correspondence with fitness and diet

**Figure 3:**
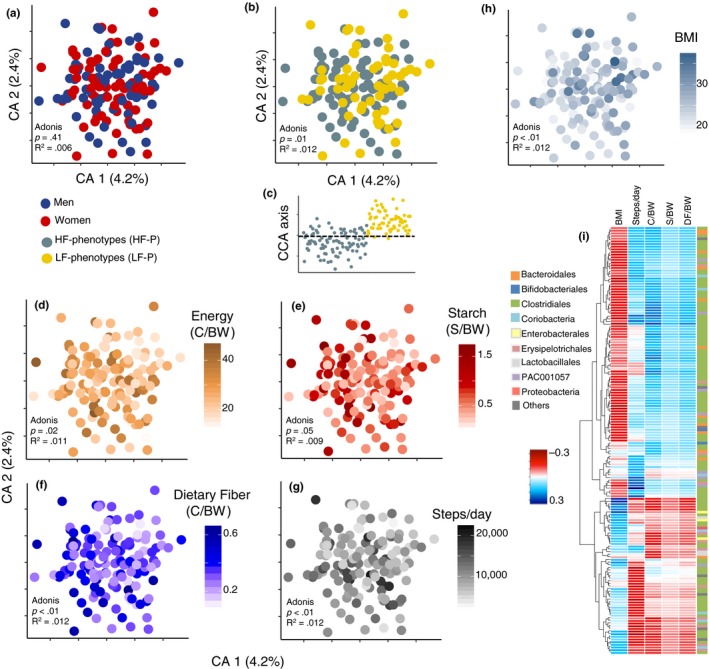
Dietary intake and fitness phenotypes are linked with species‐level GM patterns. (a) Gut microbiota (GM) composition determined through Correspondence Analysis of 16S rRNA gene (V3‐region) amplicons (summarized zOTUs at species level) determined in the stool samples of the study participants. (b) Correspondence Analysis revealed compositional GM differences between fitness phenotypes. (c) Constrained Correspondence Analysis (CCA) displays discrimination of phenotypes based on permutational test (*p* = .03, explained variance = 3.2%). (d) Correspondence Analysis of GM composition depicting gradients of total energy consumption (Cal kg body weight^−1^ day^−1^), intake of (e) starch (g kg body weight^−1^ day^−1^) and (f) dietary fiber (g kg body weight^−1^ day^−1^), (g) steps per day, and (h) BMI. (i) Regularized canonical correlation (rCC) analysis depicting the relationship between gradients of energy consumption, starch and dietary fiber intake, steps per day and BMI, and variations in the abundance of GM members. Heatmap displays the correlation of 161 species with a minimum correlation coefficient of |0.2|*r* from 1st to 3rd components. Species are depicted based on family‐level phylogeny. Figure S3 displays taxonomy at species level, as well as correlations per canonical axis and explained variance between GM composition and lifestyle covariates derived from rCC analysis. GM profiling was based on 11.3 million reads derived from the 16S rRNA gene V3‐region with an average of 116,476 (48,872 *SD*) sequences per subject. Adonis tests were performed on Bray–Curtis distances. GM data matrix: [obj × vars] = [184 × 874].

The analysis of amplicon‐sequencing data generated 10,084 zOTUs (sequence variants) summarized over 875 cumulative species (species richness) and eight core species (defined as being present in all recruited subjects) (Figure S2a) with a relative abundance ranging between 18% and 84% (Figure S2b). Between sexes, no significant differences in beta‐diversity (Figure 3a) and alpha‐diversity (Figure S2c) were observed. Furthermore, regardless of sex, the study participants were characterized by higher relative abundance of, for example, Lachnospiraceae spp., *Akkermansia* spp*.*, *Blautia* spp., along with reduced proportions of *Bacteroides* spp. (Figure S2d) as compared to the community‐dwelling group of older adults recruited for the Irish ELDERMET study (Claesson et al., 2012). This may reflect differences associated with dietary habits, age [mean age: baseline‐CALM 70 ± 4 years, ELDERMET 78 ± 8 years], and geographical location.

A substantial higher alpha‐diversity (*p* = .06, Observed Species) was observed (Figure S2c) among HF phenotypes compared to LF phenotypes, as well as weak but significant (*p* < .05) correlations of observed species with BMI, energy, and starch intake (Figure S2e–g). Correspondence analysis and analysis of variance (Adonis) on Bray–Curtis (weighted beta‐diversity) distance metric calculated from species‐level abundance showed significant correspondence (*p* = .04) and dissimilarities (*p* = .01) in GM composition in connection with the two physical phenotypes (Figure 3b,c).

Also, GM composition was clearly associated with (*p* < .05) gradients of energy consumption (Figure 3d), starch (Figure 3e), dietary fiber (Figure 3f) steps per day (Figure 3g), and BMI (Figure 3h) reflecting fitness phenotypes. Using regularized canonical correlation (rCC) analysis associations between those lifestyle covariates (e.g., dietary factors and physical activity) with 161 microbial species were disclosed (Figure 3i, Figure S3) explaining <5% and 13% of the total variance of the microbiota and lifestyle covariates, respectively (Figure S3a,b). Increased intake of energy, starch, dietary fiber, as well as steps per day correlated positively with the relative abundance of up to 103 of those species (e.g., higher Bifidobacteriales abundance) and correlated negatively with BMI (e.g., Proteobacteria being signatures for high BMI) (Figure 3i, Figure S3b).

### 2.5 Host metabolic state in relation to fitness and dietary intake

Untargeted gas chromatography–mass spectrometry (GC‐MS) metabolomics of human fecal extracts and blood plasma, as well as targeted SCFA analysis using GC‐MS generated a total of 304 analytes (181 analytes in the fecal and 123 analytes in the plasma metabolome). Nearly half of the metabolites variables were identified, either at level 1 or level 2 according to the Metabolomics Standards Initiatives (Sumner et al., 2007). These metabolites were monosaccharides, amino acids, organic acids, sterols and long‐, and short‐chain fatty acids. In addition, 31 biomarkers for immunological function, renal and liver function, as well as glucose and lipid metabolism were acquired through blood clinical profiling.

**Figure 4:**
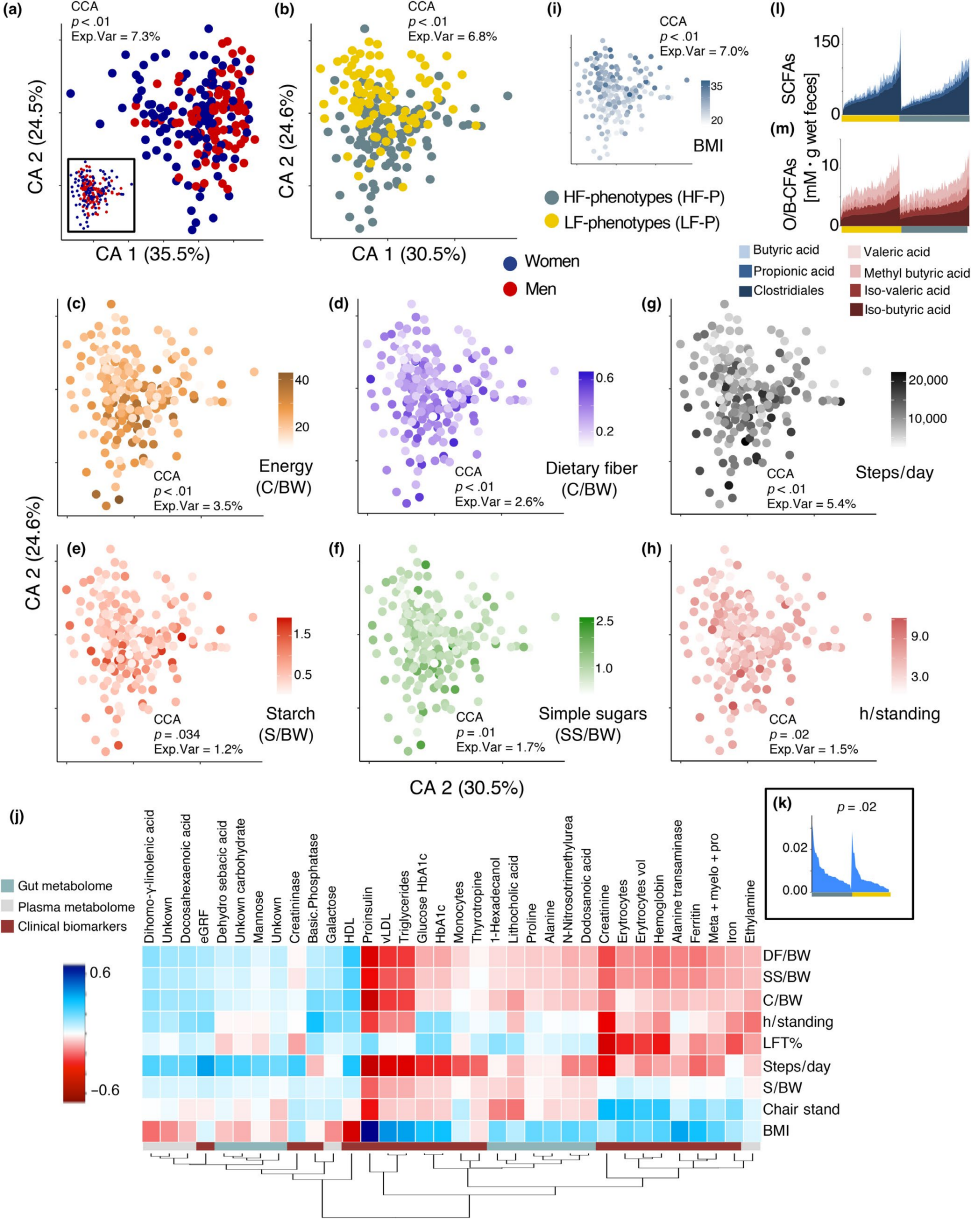
Profiling of host metabolome in relation to dietary intake. (a) Correspondence Analysis on combined fecal, plasma metabolomes and clinical biomarkers of the study participants. Significant differences due to sex were determined with constrained correspondence analysis (CCA). Inset shows a partial Correspondence Analysis after conditioning for the cofounding effect of sex. (b) Correspondence Analysis discriminates compositional differences in metabolomic profiles between fitness phenotypes. (c) Correspondence Analysis of metabolites in relation to total energy consumption (Cal kg body weight^−1^ day^−1^), intake of (d) dietary fiber (g kg body weight^−1^ day^−1^), (e) starch (g kg body weight^−1^ day^−1^) and (f) simple sugars (g kg body weight^−1^ day^−1^), (g) steps per day, (h) hours standing, and (i) BMI. (j) Regularized canonical correlation (rCC) analysis showing the relationship between gradients of energy consumption, dietary fiber, starch and simple sugar intake, steps per day, hours standing and BMI, with variations in metabolome composition. Heatmap displays the correlation of 34 clinical/metabolome variables with a minimum correlation coefficient of |0.2|*r* from 1st to 4th components. Figure S4 shows correlations per canonical axis as well as explained variance between metabolome composition and lifestyle covariates derived from rCC analysis. (k) Significantly (*t* test, *p* = .02) different relative distributions in enterolactone determined in fecal samples of HF and LF phenotypes. (l,m) Range of fecal SCFAs and O/B‐CFAs concentrations sorted according to fitness phenotype. Metabolome data matrix: [obj × vars] = [184 × 335]

Correspondence analysis on the combined metabolome blocks showed weak discrimination of sexes (Figure 4a) and pronounced discrimination between fitness phenotype (Figure 4b) based on their metabolic profile. Variations in metabolome composition corresponded clearly (*p* < .05) with energy intake and consumption of dietary fiber, starch, simple sugars (Figure 4c–f), as well as steps per day and hours‐standing‐per‐day (Figure 4g,h, including stratifying variables: BMI (Figure 4i), chair stand and LST%, Figure S4a,b). Likewise, rCC analysis showed significant associations between lifestyle covariates and 34 clinical/metabolic variables (Figure 4j), explaining 9% and 15% of the total variance of the metabolome and lifestyle covariates, respectively (Figure S4c). The strongest associations (>|0.2|*r*) were observed for 19 clinical biomarkers, 10 gut metabolites, and five plasma metabolites (Figure 4j). Increased intake of energy, starch, dietary fiber (or dietary covariates), as well as steps per day correlated positively with mono‐ and di‐saccharides and negatively with amino acids (Pro, Ala, Trp), glucose metabolism parameters (proinsulin, glucose HbA1c, HbA1c), lipid metabolism (triglycerides, vLDL) and renal function (creatinine, inversely to estimate glomerular filtration rate (eGFR)) measurements, primary bile acids (lithocholic acid), and N‐Nitrosotrimethylurea (Figure 4j). Moreover, a higher proportion of enterolactone in the fecal metabolome of HF subjects were also found (Figure 4k). Remarkably, the concentrations of SCFA as well as other/branched‐chain fatty acids (O/B‐CFA) in the fecal samples did not differ according to phenotypes (*p* > .13) or dietary intake factors (Figure 4l,m).

### 2.6 Dietary intake, gut microbiota, and metabolic signatures explain fitness levels independently from physical activity

**Figure 5:**
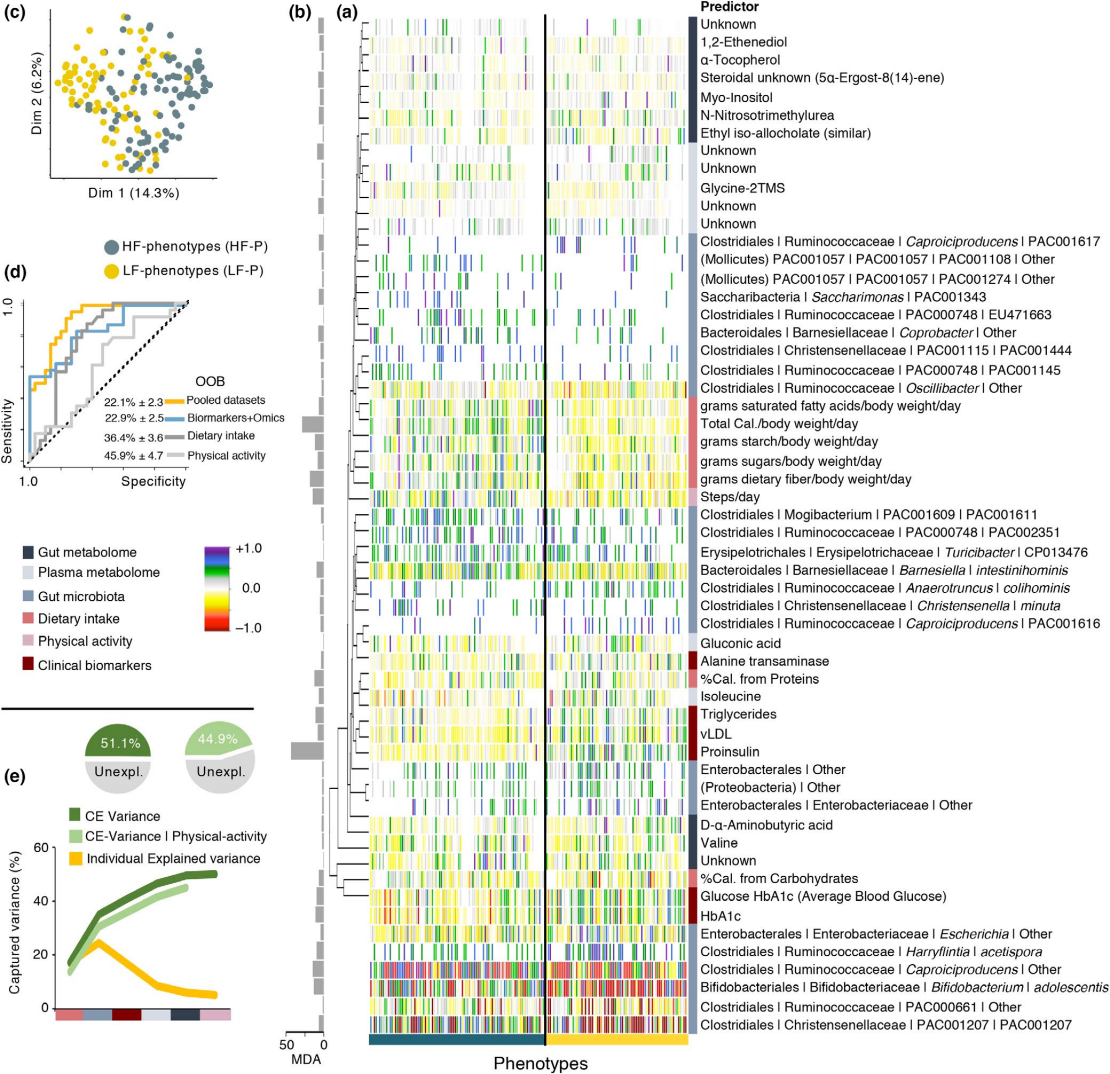
Signatures discriminating physical phenotypes. (a) Heatmap displaying mean centered normalized abundance of 55 features selected using Random Forest toward discrimination of phenotypes and (b) their importance as determined on the basis of Mean Decrease in Accuracy. (c) Multidimensional scaling plot discriminates subjects' phenotype based on the selected features. (d) ROC curves and out‐of‐bag error rate (OOB) for Random Forest classifier based on the selected variables, for combined datasets (all selected features), GM and metabolome, dietary intake, and physical activity. (e) Captured variance for fitness variables (BMI, chair stand, and LST%) as a function of selected features through redundancy analysis (RDA). Individual Explained Variance displays the size effect of a given dataset, CE variance represents the cumulative explained variance and CE variance | physical activity shows the accumulative explained variance conditioned by physical activity. Pie charts summarize the total proportion of explained variance before and after conditioning for physical activity. Data matrix: [obj × vars] = [181 × 56]

Characterization of subjects after variable selection based on Random Forest and backward elimination procedure selected 55 variables (Figure 5a,b) that discriminate the two phenotypes with a high level of accuracy (Figure 5c,d). The features included 25 bacterial species belonging to seven bacterial orders (Clostridiales, Saccharibacteria, Bacteroidales, PAC001057, Enterobacterales, Erysipelotrichales, and Bifidobacteriales), seven dietary components (energy, saturated fatty acids, simple sugars, starch and dietary fiber intake, and energy derived from proteins and carbohydrates), and five clinical biomarkers (alanine transaminase, triglycerides, vLDL, fasting proinsulin, average blood glucose/HbA1c). In addition, seven plasma metabolites (amino acids and organic acids), ten fecal metabolites (sugar alcohols, amino acids, primary bile acids, and urea) and physical activity (steps per day) were also tabbed (Figure 5a).

Discrimination of the two phenotypes based on all the selected features (combined datasets) had the highest level of accuracy (22% out‐of‐bag error rate, OOB), followed GM and clinical/metabolome features (23% OOB), dietary intake (36% OOB), and physical activity parameters (46% OOB) (Figure 5d). Through redundancy analysis (RDA), the effect of the selected variables (within blocks) on the stratifying variables showed that GM had the largest explanatory power (24.7%), followed by dietary intake (17.3%), clinical biomarkers (16.8%), gut metabolome (8.8%), plasma metabolome (6.2%), and physical activity (5.2%) (Figure 5e). Notably, the cumulative explained variance conferred by the pool of selected features reached 50.1%, and even after conditioning the effect of physical activity over the stratifying variables, the cumulative explained variance reached up to 44.9% (Figure 5f).

## 3 DISCUSSION

The number of older adults over the age of 65 will increase by more than 50% worldwide over the next three decades (NIH, 2011), potentially with huge implications for the health and economy of the implicated individuals and society as a whole. With this, understanding the physical mechanisms and lifestyle conditions linked to fitness and independence in older adults becomes a relevant field of research.

Despite the homogeneity of the recruited subjects (all nonfrail and without serious disease) noticeable significant differences in fitness level was observed and based on noncollinear ABPm variables (chair‐rise test, BMI and DXA scan‐based body composition) resulting in two fitness phenotypes (LF and HF) that differed in dietary, GM, host metabolome signatures and physical activity.

In regard to the dietary intake, HF subjects were characterized by a higher consumption of foods of plant origin as also reflected by their higher levels of total carbohydrates (i.e., starch, simple sugars) and dietary fiber, accompanied by a higher adherence to the recommended intake of carbohydrates and dietary fiber intake given by the Nordic Nutrition Recommendations (Nordic Council of Ministers, 2012). These differences were observed in spite of the methodological limitations of 3d‐WFR to capture long‐term variability (Yang et al., 2010). Furthermore, whether awareness of dietary guidelines influenced the selection of dietary choices in the study participants remains to be investigated, but it is worth mentioning that HF subjects consider healthy food as an important component in their life as also described by Schacht et al. (2019). Furthermore, the nutrient intake recorded in our study population is highly comparable to that reported for a representative sample population of older adult community‐dwelling Danes as well as for community‐dwelling Western elderly in general (Schacht et al., 2019). This indicates that the food intake generally is comparable in our study population compared to elderly community‐dwellers in Denmark and other Western countries.

The GM community and host metabolome clearly discriminated between the HF and LF phenotypes and was largely associated with the consumption of total energy, and plant‐derived nutrients (such as starch and dietary fibers as well as enterolactone, all being higher in HF subjects). A number of features (Figure 5a) selected from GM, host metabolome, dietary intake, and daily physical activity were able to strongly discriminate and explain variation between phenotypes, thereby indicating their strong association with physical function. Daily physical activity showed the lowest power toward phenotypic differentiation (in spite of the high validity of the method for activity monitoring (Dowd et al., 2012)) and explaining only 5% of the phenotypic variance. Albeit conditioning for physical activity, the remaining set of selected features explained up to 45% of the total variance of the stratifying variables. In particular, dietary intake (17% of explained variance), GM composition (24%), and host metabolome (25%) signatures are important drivers of phenotypic differentiation (Figure 5) and also described in animal models (Fujisaka et al., 2018). Accordingly, HF subjects showed a higher proportion of GM members commonly known for their protective roles, such as *Bifidobacterium adolescentis* and *Christensenella* species (Goodrich et al., 2014), and whose abundance corresponded negatively with glucose and lipid metabolism biomarkers (proinsulin, HbA1c, vLDL, triglycerides). Contrarily, LF phenotypes had increased levels of these biomarkers and a higher relative abundance of pro‐inflammatory microbial members in the gut, as for example Enterobacterales (Fei & Zhao, 2013; Khan, Nieuwdorp, & Bäckhed, 2014). Similar observations have been reported in another cohort of Danish adults (with an age range of 20–65 years), where a reduced abundance of several members of Christensenellaceae and Ruminococcaceae families corresponded with increased levels of proinsulin, HbA1c, triglycerides, and C‐reactive protein (Allin et al., 2018).

Short‐chain fatty acids derived from GM activity have been identified as signaling molecules responsible for maintenance of the integrity of colonic epithelium, glucose homeostasis, lipid metabolism, and appetite regulation (Morrison, Preston, Morrison, & Preston, 2016). Claesson et al. (2012) reported higher SCFA concentrations (acetate, butyrate, and propionate) in the fecal metabolome of older adults living as community‐dwellers compared to frail individuals living in residential care. Moreover, decreasing concentrations of these SCFAs were associated with advanced levels of frailty given by diet and specific transitions in GM composition (Claesson et al., 2012). However, in the present study no correlations between fecal SCFA and O/B‐CFA concentrations with neither macronutrient distribution or fitness phenotype were found. This suggests that levels of physical function amidst healthy older adults may not be primarily dependent upon changes in the production of these compounds. Instead, this could be due to signals of glucose metabolism deterioration as reflected by significantly (*p* < .001) higher proinsulin levels and higher average blood glucose (determined by HbA1c‐levels) in the LF phenotypes (1/116 HF and 20/91 LF subjects had higher than normal ranges of proinsulin (chi‐squared *p* < .001), 10/116 HF and 30/91 LF had higher ranges than those recommended for HbA1c (Gardner & Shoback, 2011) (chi‐squared *p* < .001), see Table S3). High concentrations of proinsulin indicate high‐insulin secretion and hence diminished peripheral insulin sensitivity resulting in a number of metabolic conditions, compromising muscle strength and physical performance (Segerström et al., 2011). Proinsulin was the most important feature of phenotype discrimination and corresponded inversely with the abundance of *Bifidobacterium adolescentis* and several species of *Christensenella,* and Ruminococcaceae (Figure 5a), strongly indicating that GM‐proinsulin interactions could be mediators of fitness phenotype. *Bifidobacterium* species (including *B. adolescentis*) have previously been described as promoters of adiponectin and decreasing expression of interleukin‐6, both playing prominent roles in metabolic derangements associated with glucose regulation and fatty acid oxidation (Aoki et al., 2017; Straub & Scherer, 2019; Su et al., 2015). *Christensenella minuta* (another Clostridiales member) is enriched in individuals with low BMI and has been demonstrated to reduce weight gain and adiposity in mice (Goodrich et al., 2014). Furthermore, while playing a protective role against inflammation, some Clostridiales members act as promoters of regulatory T cells by interacting with toll‐like receptors 2 (TLR2) on intestinal epithelial cells (Kashiwagi et al., 2015). Contrarily, species of Enterobacterales have been consistently linked with insulin resistance and inflammatory responses (Fei & Zhao, 2013; Khan et al., 2014), and by means of cell epitopes (i.e., LPS) they interact with TLRs triggering pathogen recognition, low‐grade inflammation (Franceschi & Campisi, 2014) and fat accumulation in adipose tissue that ultimately influence muscle strength (Boulangé et al., 2016).

In summary, our findings suggest that dietary patterns underlie mechanisms of physical phenotype differentiation among well‐functioning community‐dwelling older adults, particularly as a driver of GM and glucose metabolism interactions. We are aware of the cross‐sectional nature of the study and the possibility of reverse causation effects that may limit any possible attempt to determine causal features governing physical fitness as outcome. However, in spite of this we identify lifestyle, microbiome, metabolic, and daily physical activity signatures able to largely explain physical fitness, while revealing factors that could be considered as therapeutic targets in future interventions. More specifically, our study emphasizes the central role of diet toward the onset of physical deterioration and its implications prior to clinical manifestations of frailty, for example, muscle composition and diminished strength (Xue, 2011). Many of the dietary, GM, and metabolomic signatures seen in frail older adults (Bodogai et al., 2018; Claesson et al., 2012; Kong et al., 2016; Lustgarten et al., 2014) are already evident in the nonfrail, community‐dwelling older adults of low‐fitness of this study, pointing at the importance of early intervention strategies, also in this age group. Thus, in view of these findings, developing strategies to improve awareness and adherence to dietary recommendations (complying with dietary reference intakes or even with personalized nutrition (Zeevi et al., 2015)), targeting the regulation of GM and host metabolome interactions, can open opportunities to delay the comorbidities of aging.

## 4 EXPERIMENTAL PROCEDURES

### 4.1 Study participants

Two hundred and seven subjects (65+ years of age) were selected at baseline of the CALM intervention project following previously described criteria (Bechshøft et al., 2016). Participants were not allowed to take part in any organized sports or resistance training more than once a week, did not suffer from defined metabolic‐, tissue‐, or gastrointestinal disorders, nor were prescribed antibiotics 3 months prior sample collection and enrollment. Medication records of participants were documented and summarized over blood pressure lowering, statins, proton‐pump inhibitors, antihistamine, anti‐inflammatory medications, and dietary supplements (including fish oil, vitamins, and calcium).

### 4.2 Ethics approval and consent to participate

Procedures of the CALM project (Clinical Trials NCT02115698) were approved by the Danish Regional Ethical Committees of the Capital Region (J‐nr. H‐4‐2013‐070) and performed according to the Declaration of Helsinki II and the experimental designed followed as previously described (Bechshøft et al., 2016). Upon inclusion, all subjects gave their written informed consent to participate also in accordance with the Declaration of Helsinki II.

### 4.3 Samples and metadata collection

At baseline, study participants completed a 3‐day weighted food record where total food and beverage intake were registered for 3‐consecutive days (Wednesday to Friday). The dietary information collected in these food records was then typed into the electronic dietary assessment tool, VITAKOST™ (MADLOG APS), which uses the Danish Food Composition Databank (version 7.01) to estimate individual energy and macronutrient intake.

Fecal and blood plasma samples were collected and handled according to the following procedures: (a) fecal samples were kept at 4°C for maximum 48 hr after voidance and stored at −60°C until further use; (b) overnight‐fasted‐state (OFS) plasma samples were collected and deposited in heparin, centrifuged at 3,000 *g* for 10 min at 4°C, and then stored at −60°C.

For screening of blood biomarkers, the following tests were performed: complete blood count (CBC), proinsulin‐C‐peptide (P‐CP), glycosylated hemoglobin (HbA1c), coagulation factor, estimate glomerular filtration rate (eGFR), thyroid‐stimulating hormone (TSH), and iron–ferritin test determined as previously described (Bechshøft et al., 2016). On average biohumoral measurements showed an interserial uncertainty range of 3%–15%. For anthropometric and functional capacities, height (cm) and body weight (kg) in OFS were measured. Average fast‐pace gait speed was measured on an indoor 400 m horizontal track. Number of chair stands in 30 s from a standard table chair was recorded. Relative leg‐soft‐tissue fat% (LST%) was determined as an estimate of leg‐soft‐tissue fat‐free and fat mass based on a dual‐energy X‐ray absorptiometry (DXA) scan (Lunar iDXA Forma with enCORE Software Platform version 15, GE Medical Systems Ultrasound & Primary Care Diagnostics) performed on participants following standardization of subject presentation and positioning on the scanning bed, as well as manipulation of the automatic segmentation of regional areas of the scan results (Nana, Slater, Stewart, & Burke, 2015).

### 4.4 Quantitative questionnaires on food habits

Quantitative questionnaires contained information on food habits, perceptions and preferences, as well as information about lifestyle changes and dietary habits over the life course (Bechshøft et al., 2016).

### 4.5 GM and metabolomics

Procedures for profiling and process GM and metabolomics data are described in Supplementary Methods.

### 4.6 Statistical analyses

Stratification of individuals was based on ABP measurements using the variables described in Table S1. Collinear variables were initially removed, leaving chair stand [30 s‐test]), DXA scans (leg‐soft‐tissue fat% determined in both legs) and BMI as features with a variance inflation factor (VIF) < 2 and *r‐*coefficient < .5. Subjects were divided according to sex, and a hierarchical clustering analysis of principal component analysis (Husson, Josse, Lê, & Mazet, 2019) was performed on the selected variables (100 iterations).

For univariate data analyses, pairwise comparisons were carried out with unpaired two‐tailed Student's *t* test, Pearson's coefficient was used for determining correlations and chi‐square test for evaluating groups distributions. For multivariate data analyses, the influence of covariates (e.g., dietary components and BMI) on data blocks (GM and metabolome) were assessed with (Constrained‐) Correspondence Analysis with permutation tests (1,000 permutations), as well as analysis of variance using distance matrices (Adonis test, 999 permutations) on Bray–Curtis distances (implemented in the *Vegan* R package (Oksanen et al., 2019)).

Correlation of covariates with the same datasets were determined with regularized canonical correlation (rCC) analysis using the *mixOmics* R package (González, Cao, Davis, & Déjean, 2012). Regularized canonical correlation was crossed‐validated (leave‐one‐out approach) with grids (lambda 1 and 2) of 0.05–1.0 and a length of 20.

Feature selection for combined datasets was performed with Random Forest. Dataset was randomly divided 200× (200 subsets) into training (70%) and test sets (30%), keeping this proportion over the number of subjects within each fitness group for every split. For a given training set, the *party* R package (Hothorn, Hornik, Strobl, & Zeileis, 2019) was run for feature selection using unbiased‐trees (cforest_unbiased with 6,000 trees) and AUC‐based variable (varimpAUC with 100 permutations), and subsequently, the selected variables were used to predict (6,000 trees with 1,000 permutations) their corresponding test set using *randomForest* R package (Liaw, 2018). The features derived from the subset with a prediction rate within 1 *SD* above the mean prediction (based on the 200 subsets) were selected and subsequently subjected to sequential rounds of feature selection (following the same tuning of unbiased‐trees and AUC‐based variable) until prediction could no longer improve. Variation partitioning of stratifying variables (BMI, CS, and LST%) based on selected features derived from the different datasets (i.e., GM, diet, host metabolome, physical activity) was performed using redundancy analysis (RDA) (Oksanen et al., 2019). All statistical analyses were performed in R versions ≤3.6.0.

## CONFLICT OF INTEREST

None declared.

## AUTHORS' CONTRIBUTION

Conceptualization: D.S.N., J.L.C., S.B.E., L.H., and A.P.J.; Methodology: D.S.N., J.L.C., S.B.E., L.H., A.P.J., A.J.L., T.J., S.R., and R.L.B.; Formal Analysis: J.L.C., B.K., Ł.K., D.S.N., S.B.E., and L.H; Writing—Original Draft: J.L.C. and D.S.N.; Investigation, review and editing: all authors; Visualization: J.L.C.and D.S.N.; Supervision: D.S.N., S.B.E., and L.H.; Funding Acquisition: D.S.N., S.B.E., L.H., and A.P.J.

## Data Availability

Sequence data are available at the European Nucleotide Archive, accession number ENA: PRJEB33008 ([dataset] Castro‐Mejía et al., 2019). The remaining data that support the findings of this study are available on request from the corresponding authors. The data are not publicly available due to privacy or ethical restrictions.
