# Supplementary material for: Physical fitness in community‐dwelling older adults is linked to dietary intake, gut microbiota, and metabolomic signatures
Source: Aging Cell. 2020 Jan 22;19(3):e13105. doi: 10.1111/acel.13105 (PMC7059135; doi:10.1111/acel.13105)
Supplement: Supplementary file 1 [file ACEL-19-e13105-s001.pdf]

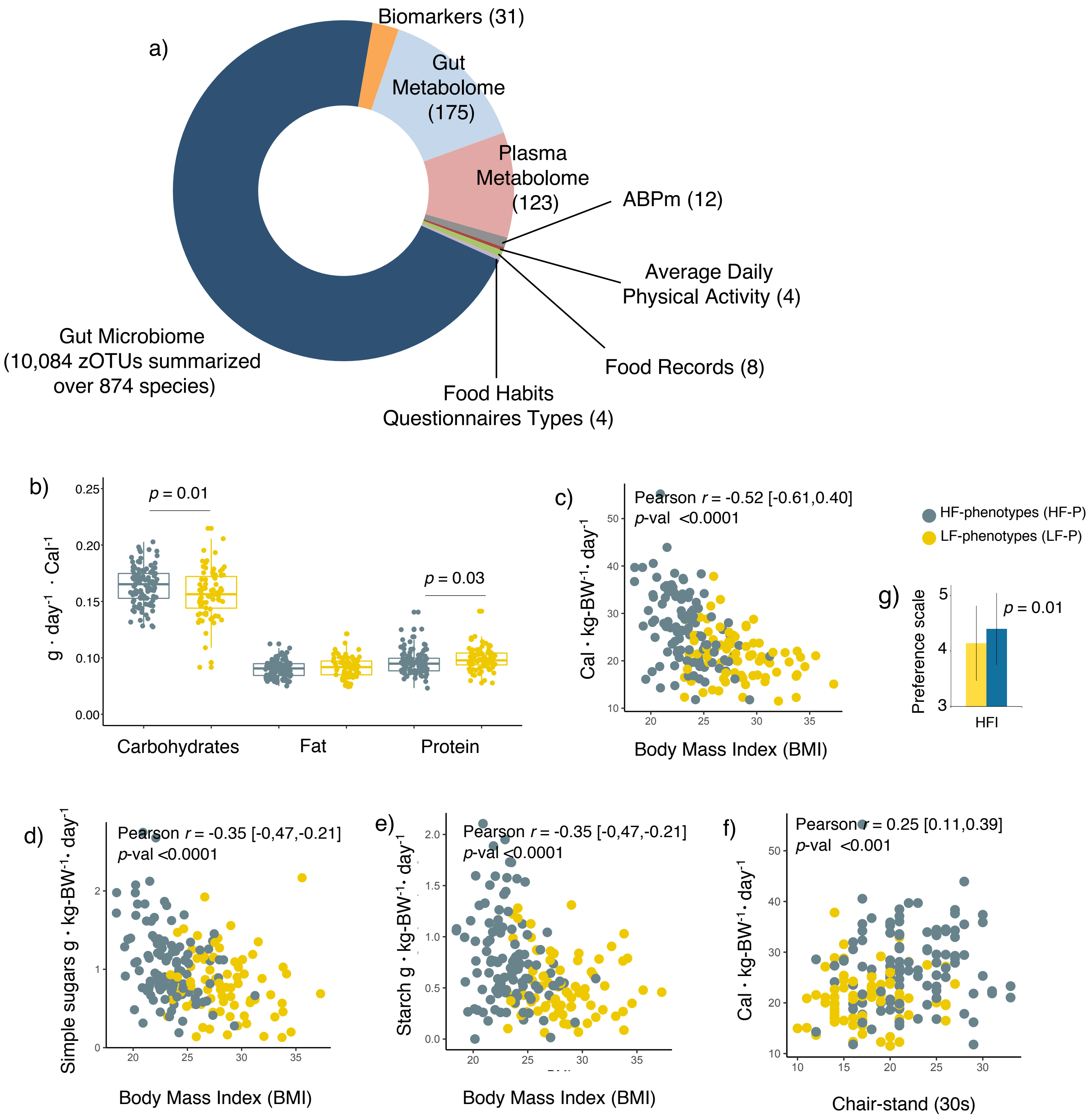

Supplementary Fig. 1. Subjects characterization and dietary intake

(a) Ring chart displays the proportion of variables used for every category at which individuals were characterized
(b) Distribution of daily macronutrient intake (g day^{-1}) normalized by the total energy intake (Cal)
(c) Correlation between energy intake (Cal · kg-BW^{-1}· day^{-1}) *vs* BMI
(d) Correlation between starch intake (g · kg-BW^{-1}· day^{-1}) *vs* BMI
(e) Correlation between simple sugars intake (g · kg-BW^{-1}· day^{-1}) *vs* BMI
(f) Correlation between energy intake (Cal · kg-BW^{-1}· day^{-1}) *vs* Chair-stand test
(g) Degree of agreement for food-choices questionnaires: healthy food in an important element of everyday life (HFI). This was evaluated on a scale of 1 – 5. 1: is not important, 5: is very important,

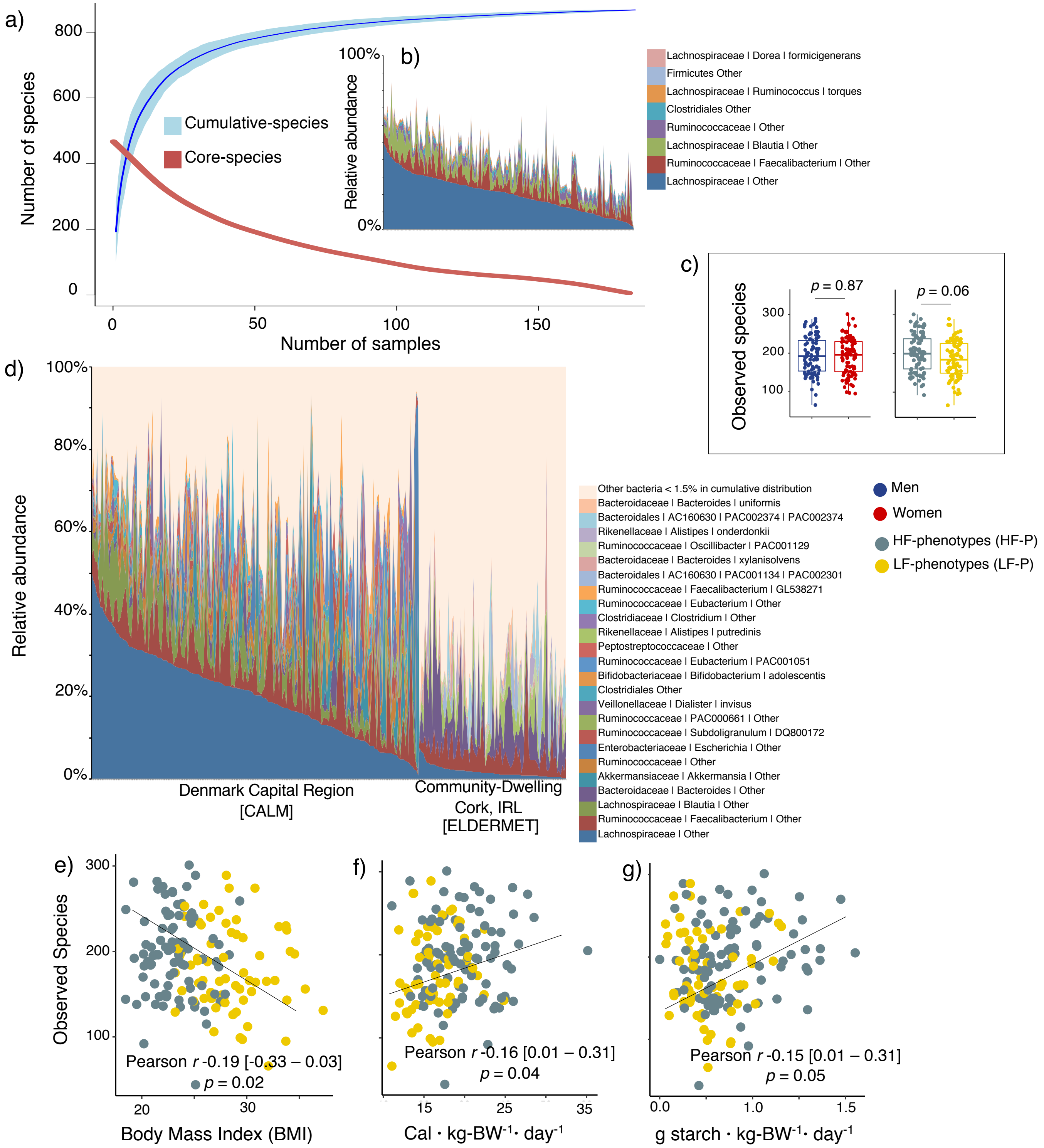

Supplementary Fig. 2. GM overview, cumulative- and core-species

(a) Cumulative- and core species comprised in the CALM study with increasing number of subjects.
(b) Relative abundance of core-species (phylotypes summarized to species level).
(c) Alpha diversity (of summarized zOTUs at species level) between sexes (p = 0.42) and phenotypes (p = 0.04) determined by Monte Carlo permutation (100) test.
(d) Distribution of species across the subjects of the CALM intervention (16S rRNA v3 region) and the community-dwellers of the ELDERLMET study (16S rRNA v4 region, 454 pyro-sequencing). The publically available sequencing data from the ELDERMENT study were retrieved and analyzed using the parameters described in methods.
(e) Correlation between observed species *vs* BMI
(f) Correlation between observed species *vs* Energy intake (Cal · kg-BW^{-1}· day^{-1})
(g) Correlation between observed species *vs* Simple sugars intake (g · kg-BW^{-1}· day^{-1})

Supplementary Fig. 3. rCC analysis between GM and lifestyle components

(a) Canonical correlation within 1st to 3rd components as well as explained variance between GM composition and lifestyle covariates.
(b) Associations between GM species and lifestyle covariates with a minimum correlation coefficient of |0.2|

a)

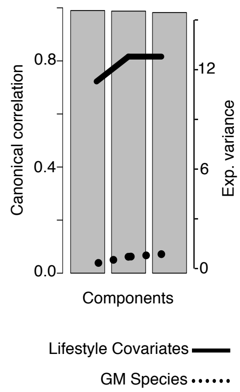

b)

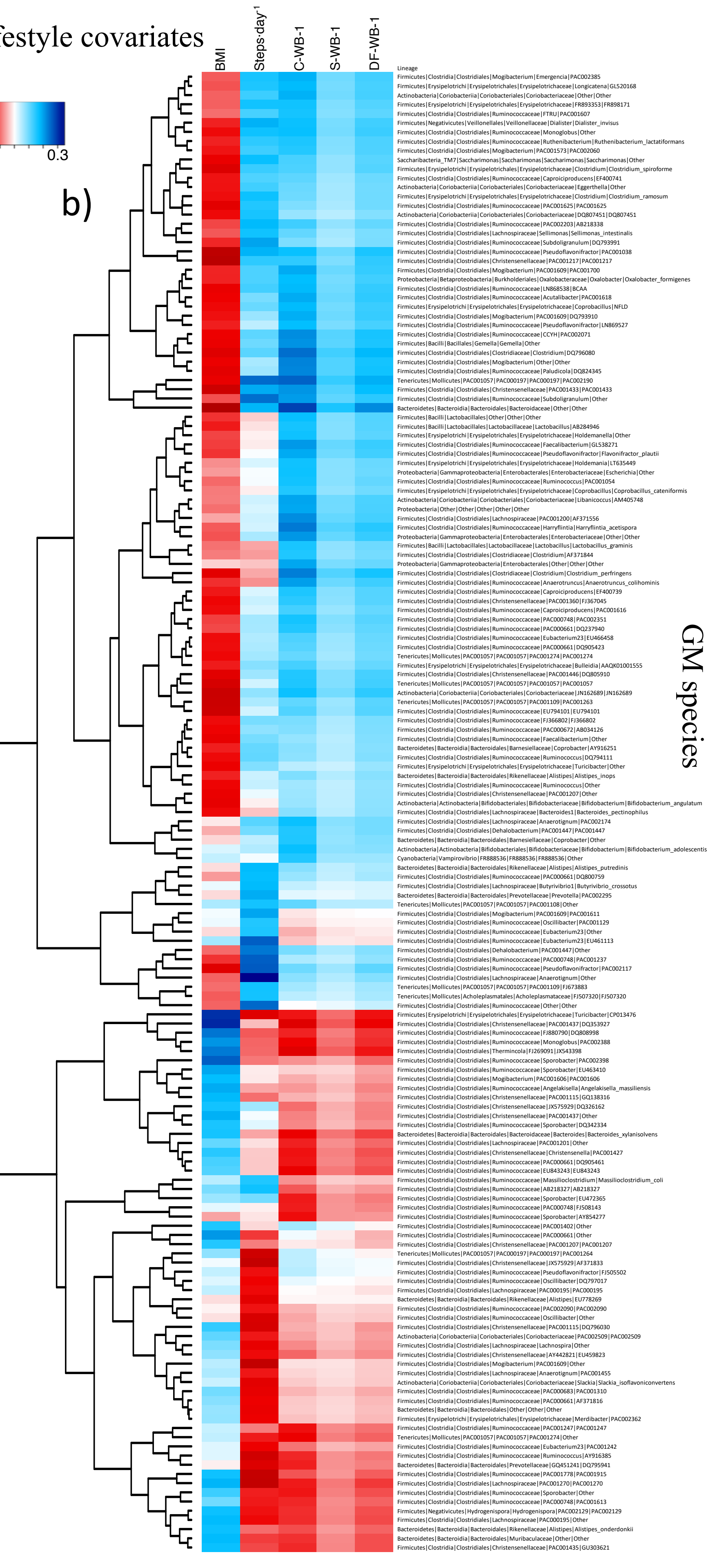

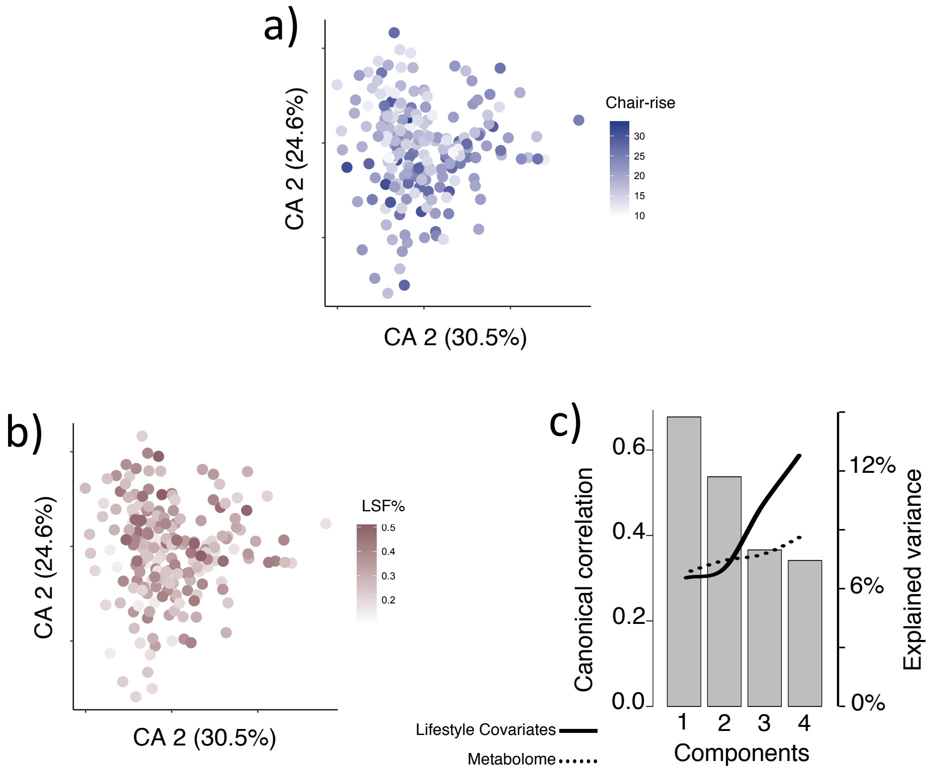

Supplementary Fig. 4. Metabolome correspondence and correlation

(a) Correspondence Analysis of metabolome in relation to chair-stand test and (b) LSF%
(c) Canonical correlation within 1st and 4th components as well as explained variance between metabolome profiling and lifestyle covariates.
