# Supplementary material for: Physical fitness in community‐dwelling older adults is linked to dietary intake, gut microbiota, and metabolomic signatures
Source: Aging Cell. 2020 Jan 22;19(3):e13105. doi: 10.1111/acel.13105 (PMC7059135; doi:10.1111/acel.13105)
Supplement: Supplementary file 5 [file ACEL-19-e13105-s005.docx]

## Fecal DNA extraction, 16S rRNA-gene amplicon sequencing

Fecal samples were thawed at 4°C, re-suspended in ultrapure water (1:2 feces/water) and homogenized in filter bags for 1 min at high speed (Lab Seward, BA7021). 1.5 ml of the fecal slurry was centrifuged at 13,000×g for 10 min at room temperature and ~200 mg of the fecal pellet was used for DNA extraction using the PowerSoil® DNA Isolation Kit (MOBIO Laboratories, Carlsbad, CA, USA), basically following the instructions of the manufacturer, but with minor modifications to increase lysis of bacterial cells: prior DNA extraction, samples were placed into the PowerBead tubes and heat treated at 65°C for 10 min and then at 95°C for 10 min. Subsequently, solution C1 was added and bead-beating performed in FastPrep (MP Biomedicals, Santa Ana, CA, USA) using 3 cycles of 15 s each, at a speed of 6.5 m s^-1^. The remaining DNA extraction procedure followed the manufacturer’s instructions. Gut prokaryotic composition was determined by NexSeq 500 based 16S rRNA gene-amplicon sequencing of the V3 region amplified using primers designed with adapters for the Nextera Index Kit® (Illumina, CA, USA): NXt_338_F: 5’- TCG TCG GCA GCG TCA GAT GTG TAT AAG AGA CAG ACW CCT ACG GGW GGC AGC AG -3’ and NXt_518_R: 5’- GTC TCG TGG GCT CGG AGA TGT GTA TAA GAG ACA GAT TAC CGC GGC TGC TGG -3’. Amplification profile (1^st^ PCR), barcoding (2^nd^ PCR), amplicon library purification and sequencing were performed as previously described (Pyndt Jørgensen et al. 2014).

## Analysis of high-throughput amplicon sequencing

The raw dataset containing pair-ended reads with corresponding quality scores were merged and trimmed using the following settings, -fastq_minovlen 100, -fastq_maxee 2.0, -fastq_truncal 4, -fastq_minlen 130. Finding unique reads and deconvoluting from chimeric reads and constructing *de-novo* zero-radius Operational Taxonomic Units (zOTU) was conducted using the UNOISE pipeline (Edgar 2018) coupled to the EZtaxon 16S rRNA gene collection as a reference database (Kim et al. 2012). Downstream analyses were based on a contingency table rarefied to 17,000 sequences per sample and then normalized with cumulative sum scaling (CSS (Paulson et al. 2013)).

## Metabolomics

### Untargeted metabolomics of fecal slurries

1 ml fecal homogenate (as described above) was mixed with 1 ml of Sterile PBS (5.7 mM Na_2_HPO_4_, 24.3 mM NaH_2_PO_4_, 450 mM NaCl, pH 7.4), frozen in liquid nitrogen and freeze-dried overnight. Twenty mg of each freeze-dried sample were re-suspended in 1 ml of 99.98% methanol (containing 10 ppm palmitic-acid methyl ester and 10 ppm sorbitol as an internal standards), vortexed and centrifuged for 30 min at 12,000×g at 4°C. Fifty µl of the supernatant were then dried using a ScanVac (Labogene, Lynge, Denmark) at 1,000 rpm for 3 h at 40°C. Immediately after drying, samples were sealed with air tight magnetic lids into 2.0 ml GC-MS vials and derivatized in two steps using a Dual-Rail MultiPurpose Sampler (MPS) (Gerstel, Mülheim an der Ruhr, Germany), (i) addition of 10 µl of MEOX reagent (20 mg ml^-1^ Methoxiamine hydrochloride in dry pyridine) followed by agitation at 45°C for 90 min by mixing at 750 rpm, (ii) addition of 40 µl of TMS reagent, trimethylsilyl cyanide (TMSCN) (Khakimov et al. 2013) followed by agitation at 45°C for 45 min by mixing at 750 rpm. All steps involving sample derivatization and injection were automated using MPS, which was equipped with a sample agitation unit. Immediately after derivatization, 1 μl of the derivatized sample was injected into a cooled injection system (CIS4) (Gerstel, Mülheim an der Ruhr, Germany) port in splitless mode. The septum purge flow and purge flow to split vent at 2.5 min after injection were set to 25 and 15 ml min^-1^, respectively. Initial temperature of the CIS4 port was 45°C, and heated at 12°C s^-1^ to 320°C (after 30 s of equilibrium time), where it was kept for 10 min. After heating, the CIS4 port was gradually cooled to 250°C at 5°C s^-1^, and this temperature was kept constant during the run. The GC-TOF-MS setup was made combining an Agilent 7890B gas chromatograph (GC) (Agilent Technologies, California, USA) with a time-of-flight mass spectrometer, HT Pegasus TOF-MS, (LECO Corporation, Saint Joseph, USA). GC separation was performed on a Zebron ZB 5% Phenyl 95% Dimethylpolysiloxane column (30 m with I.D. 250 μm and film thickness 0.25 μm) with a 5 m inactive guard column (Phenomenex, Torrance, USA). A hydrogen generator, Precision Hydrogen Trace 500 (Peak Scientific Instruments Ltd, Inchinnan, UK) was used to supply a carrier gas at a constant column flow rate of 1.0 ml min^-1^. The initial temperature of the GC oven was set to 40°C and held for 2 min followed by heating at 10°C min^-1^ to 320°C and kept for an additional 6 min, making the total run time 36 min. Mass spectra was recorded in the range of 45–600 m/z with a scanning frequency of 10 scans sec^-1^, and the MS detector and ion source was switched off during the first 6.3 min of solvent delay time. The transfer line and ion source temperature were set to 280°C and 250°C, respectively. The mass spectrometer was tuned according to manufacturer’s recommendation using perfluorotributylamine (PFTBA). MPS and GC-TOF-MS were controlled using vendor software Maestro (Gerstel, Mülheim an der Ruhr, Germany) and ChromaTOF (LECO Corporation, Saint Joseph, USA), respectively. Samples were randomized prior to derivatization and GC–MS analysis. In order to monitor instrument performance, a blank sample containing only derivatization reagent, a control sample (a pooled sample), and an alkane mixture standard sample (all even C10-C40 alkanes at 50 mg L^-1^ in hexane) were injected after every 10 real samples.

The raw GC-TOF-MS data was processed using Statistical Compare toolbox of the ChromaTOF software (Version 4.50.8.0) with following settings; the raw data was used without smoothing prior to peak deconvolution, baseline offset was set to 0.8, expected averaged peak width was set to 1.5 sec, signal-to-noise was set to ≥10, peak areas were calculate using deconvoluted mass spectra (DT), common *m/z* ions of derivatization products were determined as 73, 75, and 147, deconvoluted mass spectra were also used for peak identification using LECO-Fiehn and NIST11 libraries. The library search was set to return top 10 hits with EI-MS match of >75% using normal-forward search and with a mass threshold of 20. Deconvoluted peaks were aligned across all samples using following settings; retention time shift allowance of <3 sec, EI-MS match of >95%, mass threshold of >25, and present in >90% of all pooled control samples.

### Targeted analysis of SCFA and O/B-CFA in fecal slurries

Analysis of SCFA and O/B-CFA was performed on 0.5 ml of fecal homogenate mixed with 1 ml of 0.3M oxalic acid (containing 2 mM of 2 ethylbutyrate (Sigma-Aldrich) as the internal standard). Samples were vortexed for 1 min, centrifuged at 20°C for 20 min at 12,000×g, followed by filtration using a 0.45 µm centrifugal filter (Millipore UFC30HV00) and the obtained aliquot was used for GC-MS analysis. The GC-MS consisted of an Agilent 7890A GC and an Agilent 5973 series MSD. GC separation was performed on a Phenomenex Zebron ZB-WAXplus column (30 m × 250 μm × 0.25 μm). A sample volume of 1 μl was injected into a split/splitless inlet at 285°C using split mode at 2:1 split ratio. Septum purge flow and split flow were set to 13 ml min^-1^ and 2 ml min^-1^, respectively. Hydrogen was used as carrier gas, at a constant flow rate of 1.0 ml min^-1^. The GC oven program was as follows: initial temperature 100°C, equilibration time 1.0 min, heat up to 120°C at the rate of 10°C min^-1^, hold for 5 min, then heat at the rate of 40°C min^-1^ until 230°C and hold for 2 min. Mass spectra were recorded in Selected Ion Monitoring (SIM) mode and m/z ions were detected at the dwell time of 50 msec: 41, 43, 45, 57, 60, 73, 74, 84. The detector was switched off during the 1 min of solvent delay time. The transfer line, ion source and quadrupole temperatures were set to 230, 230 and 150°C, respectively. The mass spectrometer was tuned according to manufacturer’s recommendation using perfluorotributylamine (PFTBA). Dilution series of SCFA standards of acetic, propionic, butyric, isobutyric, 2-methyl isobutyric, valeric and isovaleric acid (Sigma-Aldrich) were prepared in concentrations of 1.000, 0.500, 0.250, 0.125, 0.060 and 0.030 mM for the construction of standard curves for quantification. Initial inspection of the GC-MS data was performed using MSD ChemStation software (Version E.02.02.1431, Agilent Technologies, Inc., Germany). Mass spectra of SCFA were compared against the NIST11 library (NIST, Maryland, USA). SCFA peak areas were integrated from SIM chromatograms using in-house Matlab (Version. R2015a, The MathWorks, Inc., Massachusetts, USA) scripts. Two SCFA, 2-methyl isobutyric acid and isovaleric acid, co-eluted at the retention time range of 4.22-4.45 min, thus peak areas were calculated by deconvoluting these peaks using *m/z* ions 74 for 2-methyl isobutyric acid and 60 for isovaleric acid.

### Untargeted metabolomics of blood plasma

A mixture of 100 µl of plasma samples (thawed at room temperature) and 300 µl of MeOH:water (8:1, vol:vol and containing 10 ppm of sorbitol as internal standard) were vortexed (highest speed) for 1 min. Thereafter, samples were incubated at 4°C for 15 min and centrifuged at 16,000×g at 4°C for 10 min. Supernatants were passed through a 0.45 µm centrifugal filter (Millipore UFC30HV00) and 80 μl aliquots were dried into 200 μl glass inserts using a ScanVac (Labogene, Lynge, Denmark) at 40°C for 3 h at 1,000 rpm. Immediately after drying samples were sealed with air tight magnetic lids into 2.0 ml GC-MS vials and derivatized in two steps using MPS, (i) addition of 10 µl of MEOX reagent (20 mg ml-1 Methoxiamine hydrochloride in dry pyridine) followed by agitation at 65°C for 60 min by mixing at 750 rpm, (ii) addition of 30 µl of TMS reagent (TMSCN) followed by agitation at 65°C for 2 h by mixing at 750 rpm. Immediately after derivatization, 1 μl of the derivatized sample was injected into the GC-TOF-MS as described for the fecal metabolomics. Sample injection, oven and mass spectrometer parameters were similar to those for the fecal metabolomics with few modifications. The initial temperature of the GC oven was set to 40°C and held for 2 min followed by heating at 12 °C min^-1^ to 260°C, and with a rate of 30°C min^-1^ to 320°C and kept for an additional 5 min, making the total run time 27.33 min. Mass spectra was recorded in the range of 45–500 m/z with a scanning frequency of 8 scans sec^-1^, and the MS detector and ion source was switched off during the first 8.3 min of solvent delay time. The transfer line and ion source temperature were set to 290°C and 250°C, respectively. In order to monitor instrument performance, a blank sample containing only derivatization reagent, a control sample (a pooled sample), and an alkane mixture standard sample (all even C10-C40 alkanes at 50 mg L^-1^ in hexane) were injected after every 10 real samples. The raw GC-TOF-MS data was processed as described above for untargeted fecal metabolomics.

### References

Edgar, R.C. (2018) Updating the 97% identity threshold for 16S ribosomal RNA OTUs A. *Bioinformatics* 34, 2371–2375. https://doi.org/10.1093/bioinformatics/bty113

Khakimov, B., Motawia ,M.S., Bak, S. & Engelsen, S.B. (2013) The use of trimethylsilyl cyanide derivatization for robust and broad-spectrum high-throughput gas chromatography-mass spectrometry based metabolomics. *Analytical and Bioanalytical Chemistry,* 405, 9193–9205. https://doi.org/10.1007/s00216-013-7341-z)

Kim, O.S., Cho, Y.J., Lee, K., Yoon, S.H., Kim, M., Na, H., … Chun, J. (2012) Introducing EzTaxon-e: a prokaryotic 16S rRNA gene sequence database with phylotypes that represent uncultured species. *International Journal of Systematic and Evolutionary Microbiology,* 62, 716–721. https://doi.org/10.1099/ijs.0.038075-0

Paulson, J.N., Stine, O.C., Bravo, H.C. & Pop, M. (2013) Differential abundance analysis for microbial marker-gene surveys. *Nature Methods* 10, 1200–1202. https://doi.org/10.1038/nmeth.2658

Pyndt Jørgensen, B., Hansen, J.T., Krych, L., Larsen, C., Klein, A.B., Nielsen, D.S., J… Sørensen, D.B. (2014) A Possible Link between Food and Mood: Dietary Impact on Gut Microbiota and Behavior in BALB/c Mice. *PLoS One* 9, e103398. https://doi.org/10.1371/journal.pone.0103398
